# Update on Dental Luting Materials

**DOI:** 10.3390/dj10110208

**Published:** 2022-11-03

**Authors:** Gary Kwun-Hong Leung, Amy Wai-Yee Wong, Chun-Hung Chu, Ollie Yiru Yu

**Affiliations:** Faculty of Dentistry, The University of Hong Kong, Prince Philip Dental Hospital, 34 Hospital Road, Sai Ying Pun, Hong Kong, China

**Keywords:** bridge, cariology, crown, dental cements, indirect restoration, luting agents, luting cements, permanent cements, prosthodontics, resin cements, temporary cements

## Abstract

A dental luting material aids in the retention and stability of indirect restorations on the prepared tooth structure. In dentistry, clinicians are using a wide range of luting materials for the cementation of indirect restorations. Zinc oxide eugenol and non-eugenol cements, zinc phosphate cement, zinc polycarboxylate cement, glass ionomer cement and resin cements are common dental cements used in dentistry. Each luting material or cement possesses unique properties and clinical implications. An ideal luting cement should be biocompatible, insoluble, resistant to thermal and chemical assaults, antibacterial, aesthetic, simple and easy to use. It should have high strength properties under tension, shear and compression to resist stress at the restoration–tooth interface, as well as adequate working and setting times. So far, no luting material possesses all of these properties of an ideal cement. Scientists have been modifying the conventional luting cements to improve the material’s clinical performance and developing novel materials for clinical use. To achieve the best clinical outcome, clinicians should update their knowledge and gain a good understanding of the luting materials so that they can make a wise clinical decision on the material selection and obtain an insight into the development of luting cements. Therefore, the objective of this study is to provide a discussion on the physical, chemical, adhesive and aesthetic properties of common luting materials. The clinical indications of these luting materials are suggested based on their properties. In addition, overviews of the modification of the conventional luting materials and the newly developed luting materials are provided.

## 1. Introduction

A luting cement is a material that is used to attach indirect restorations to prepared tooth surfaces by filling minute voids between the restorations and the tooth structures, thereby locking the restoration mechanically to prevent dislodgement [1,2]. A luting material provides the retention of indirect restorations by providing mechanical interlocking, chemical bonding or both of them. Traditional cementation rely mostly on the frictional forces between the prepared tooth surfaces and fitting walls of restorations [3]. More contemporary materials utilize chemical and micromechanical adhesion to bond between the tooth surface, cement and restorative material [4].

Understanding the properties of the luting materials and their clinical indications is helpful to ensure the quality of the cementation. The luting materials create the seal between the restoration and the tooth. A good seal is important not only to hold the restoration in place but also to make the surface impervious to microleakage and caries. Hence, luting materials affect the longevity of indirect restorations. In addition, the luting materials are widely indicated in the cementation of crowns, inlays, onlays, veneers, multiple-unit fixed prostheses, endodontic posts and orthodontic appliances. It is essential for dental practitioners to comprehend the clinical indications of the common luting materials. Therefore, this review aims to provide a discussion on the physical, chemical, adhesive and aesthetic properties of common luting materials. The clinical indications of these luting materials are suggested based on their properties. In addition, overviews of the modification of the conventional luting materials and the newly developed luting materials is provided.

## 2. Historical Development of Common Luting Cements

Over the past 50 years, a range of new materials has been developed which are known as luting cements. However, before this period, zinc phosphate had been the only choice of material for permanent cement for almost 100 years since the late 19th century. Therefore, it is often regarded as the “gold standard” for permanent dental cements [5]. In the late 1960s, zinc polycarboxylate was introduced, which offered more options of luting material to clinicians at that time [3]. Contemporary cements shifted from the utilization of a bonding mechanism to luting (filling in the space between the tooth and the restoration). Between the 1970s and the 1980s, glass ionomer cement (GIC) and resin-modified glass ionomer cement (RMGIC) was invented. Resin cement, which was invented in the 1950s, has undergone many reformulations and improvements over the years. Owing to the increasing demand for aesthetic all-ceramic restorations, it has gained high popularity in contemporary dentistry [5]. In the 2000s, self-adhesive resin cement was developed to simplify the clinical procedure of traditional resin cements [6]. Calcium aluminate/glass ionomer cement (CaAl/GI) has a bioactivity property by creating hydroxyapatite crystals, and this was introduced in 2009 [7,8,9]. An ideal luting cement should be biocompatible, insoluble, resistant to thermal and chemical assaults, antibacterial, aesthetic, simple and easy to use. It should have high strength properties under tension, shear and compression to resist the stress at the restoration–tooth interface, as well as adequate working and setting times. So far, no luting material possesses all of these properties of an ideal cement.

## 3. Classification of the Luting Materials

The luting materials can generally be classified by their chemical compositions, bonding mechanisms or clinical indications. Luting materials can be classified as water- or resin-based luting cements based on their chemical composition. Water-based materials include zinc-oxide eugenol and non-eugenol, zinc polycarboxylate, zinc phosphate, GIC and hybrid CaAl/GI cements. Resin-based luting cements include conventional and self-adhesive resin cements. RMGIC, which has the properties of GIC and resin cements, is a mix of water- and resin-based cement.

Luting materials can also be classified as non-adhesive, chemically adhesive and micromechanically adhesive luting agents by means of a bonding mechanism. Non-adhesive luting materials achieve restoration by friction only. Chemically adhesive luting materials can establish molecular interactions with the tooth structures to form chemical bonding, whereas micromechanically adhesive materials accomplish adhesion via micromechanical interlocking between the adhesive and the tooth surfaces [10]. Non-adhesive cements include zinc oxide eugenol and non-eugenol and zinc phosphate cements. Chemically adhesive materials include zinc polycarboxylate, GIC and hybrid CaAl/GI cements. Both types of resin cement are micromechanically adhesive materials. RMGIC can be both chemically adhesive and micromechanically adhesive.

Based on the clinical indication, luting materials can be categorized into temporary or permanent cements. Temporary cements are used to retain provisional restorations, and these include zinc oxide eugenol and non-eugenol and zinc polycarboxylate [11] (Table 1).

The permanent cements include zinc phosphate, zinc polycarboxylate, GIC, RMGIC, conventional and self-adhesive resin cement and hybrid CaAl/GI cement. They are used in the cementation of definitive restorations (Table 2).

## 4. Properties and Clinical Indication of Luting Materials

The currently available luting materials include zinc oxide eugenol and non-eugenol, zinc phosphate, zinc polycarboxylate, GIC, RMGIC, hybrid CaAl/GIC and resin cements. Their properties as luting cements are summarized below, and their clinical implications are suggested in Table 3.

### 4.1. Zinc Oxide Eugenol and Non-Eugenol Cements

Zinc oxide eugenol has a two-component system and sets in an acid–base reaction. The powder contains zinc oxide, along with a minute amount of other zinc salts and abietic acids to increase the working time and strength of it. The liquid contains eugenol and acetic acid, the latter of which acts as an accelerator [14]. There are numerous advantages to using zinc oxide eugenol as a temporary cement. Its low strength allows for the easy removal of provisional crowns. It also has sedative properties to the dental pulp and a good marginal seal, and it is cost-effective [14,35]. Although several laboratory studies and a systematic review have shown that the use of eugenol-containing temporary cement does not alter the bond strength of resin [35,36,37,38], one common concern about zinc oxide eugenol is that eugenol hinders the polymerization of resin cement and leads to a reduced bond strength [15,39]. To avoid disrupting the polymerization of a composite resin, it is suggested to use zinc oxide non-eugenol as a temporary cement before the composite resin application. It uses acids such as aliphatic acid or butyric acid to substitute the eugenol in the acid–base reaction [14].

### 4.2. Zinc Phosphate

Zinc phosphate has the longest history of use in dentistry and is often regarded as the “gold standard” for permanent dental cements [5]. It is essentially made of zinc oxide and magnesium oxide powders with a liquid consisting of phosphoric acid, water and aluminum phosphate [40]. Being a non-adhesive luting agent, it does not bond to the tooth structure and restorative materials and merely acts by providing mechanical interlocking [3]. It has moderate compressive strength and a high modulus of elasticity [41,42]. Having a high mechanical strength upon setting, it is a good choice for the cementation of a cast metal post-core [43]. It can also be used to cement all-metal or metal-supported restorations with good mechanical retention results [43,44]. The disadvantages of zinc phosphate outweigh the advantages, and its use in contemporary dentistry is quite limited. Like other water-based cements, zinc phosphate sets by means of an acid–base reaction. Due to the presence of phosphoric acid, the initial pH of the setting cement is very low (<2.0 min), and this may cause sensitivity during and after cementation. Moreover, the reaction is highly exothermic and could possibly irritate the pulp. It can also undergoes discoloration and has relatively weak mechanical properties when it is compared to the contemporary cements [44]. There is also a higher chance of secondary caries occurring in zinc phosphate-cemented restorations due to its water solubility and its inability to inhibit bacterial growth [45].

### 4.3. Zinc Polycarboxylate

Zinc polycarboxylate was developed in 1968 with the goal to develop a dental cement that has the strength of zinc phosphate with the adhesiveness and biocompatibility of zinc oxide eugenol [14,46]. It consists of zinc oxide and polyacrylic acid in a powder and liquid system, which sets via an acid–base reaction in an aqueous environment. During the reaction, zinc crosslinks the polyacrylic acid chains and leads to the setting of the cement [47]. Because the carboxylic groups of the polyacrylic acid chains can chelate the calcium in hydroxyapatite, it can create a form of a chemical bond to mineralize the hard tissues [48,49]. However, the chemical adhesion of it is weak, and the retention of it is primarily mechanical [6]. The major advantages of zinc polycarboxylate are that it is less irritating to the pulp and has a higher tensile strength when it is compared to the traditional zinc phosphate. However, its compressive strength is only one-half to two-thirds of that of zinc phosphate. Its modulus of elasticity is also much lower than that of zinc phosphate. Thus, it may display significant plastic deformation upon long-term loading [10]. Moreover, it has low resistance to acidic erosion and should not be used in patients with gastric reflux problems or who frequently consume an acidic diet [50]. Inferior handling properties due to it having a high degree of viscosity and it needing a short working time (2.5 min) are also considered as its clinical disadvantages, especially when one is cementing multiple-unit restorations [13]. Therefore, despite originally being developed as a permanent cement, the main application of polycarboxylate cement today is for the long-term maintenance of provisional restorations or when maximum retention is needed for the provisional restoration, such as when the retention form of the tooth preparation or the quality of the provisional restoration is not satisfactory [2].

### 4.4. Glass Ionomer Cement

#### 4.4.1. Conventional Glass Ionomer Cement

Conventional GIC (glass ionomer cement, GIC), which is officially known as glass polyalkenoate cement, is made of calcium fluoro-alumino-silicate glass powder combined with a water-soluble polycarboxylic acid [51]. In the 1990s, GIC was modified with fine glass particles and high molecular weight anhydrous polyacrylic acid [52]. Tartaric acid is added to increase the working time of it and facilitate the setting reaction to improve the post-setting hardness [53]. After mixing, GIC sets by an acid–base reaction where the carboxyl ion of the polycarboxylic acid forms ionic bonds with aluminum and calcium ions in fluoro-alumino-silicate glass powder. Gelation of the cement begins and continues until the cement hardens [54]. On top of the mechanical retention, GIC can form molecular adhesion to the tooth structure by the chelation with calcium and phosphate ions. An important feature of GIC is fluoride release. In the reaction, fluoride is released into an aqueous environment with an initial burst over the first 24 h, and this gradually slows down to a long-term release. Owing to its fluoride-release property, the most valuable advantage of GIC is its anticariogenic property, which aids in preventing secondary caries [55]. It is also biocompatible with the dental pulp [56]. However, its strength properties are unsatisfactory. It has low flexural strength and a high modulus of elasticity, making it brittle and prone to bulk fractures [57]. Being a water-based cement, its solubility in oral fluid and microleakages are still issues [12]. Additionally, similar to zinc phosphate, the low initial pH of setting GIC can contribute to postoperative sensitivity [58,59]. Therefore, it is suggested that one uses dentine sealers, such as resin-based bonding agents for pulpal protection, when GIC is used and when the remaining dentine thickness is minimal [60].

#### 4.4.2. Resin-Modified Glass Ionomer Cement

With the desire to improve the shortcomings of conventional GIC, RMGIC was developed to combine the strength and hydrophobicity of resin with the valuable fluoride-release ability of GIC. Monomers such as hydroxyethyl methacrylate (HEMA) are added into the liquid component of GIC, along with an associated photo-sensitive initiator system [61]. RMGIC is described to be “dual-cured”, where resin monomers undergo photopolymerization upon light curing, and the GIC component is chemically cured in an acid–base reaction. The polymerized resin acts as a bridge and strengthens the material [54]. In addition to the chemical bonding that is achieved by polycarboxylates, RMGIC can also achieve micromechanical interlocking in hybridized dentine through the infiltration of the collagen network that is exposed by 10% polyacrylic acid [62]. This creates a greater bond strength and higher fracture toughness values than conventional GIC can, although the mechanical properties are still inferior to the resin cement [63]. On the other hand, the fluoride release from RMGIC follows a similar pattern to that of conventional GIC with an initial burst during the first 24 h [64,65]. Although the amount varies by product, its fluoride release potential can be comparable to that of conventional GIC, retaining the anticariogenic property of conventional GIC [66,67]. The addition of resin is not without its downside. Several studies have compared the effects of conventional GIC and RMGIC on pulpal tissues, and the results have shown that RMGIC is more cytotoxic to pulp cells [68,69,70]. Therefore, RMGIC cannot be considered to be as biocompatible as conventional GIC is [71].

#### 4.4.3. Hybrid Calcium Aluminate/Glass Ionomer Cement

Hybrid CaAl/GIC is a water-based permanent cement that is composed of calcium aluminate and glass ionomer mixed with distilled water [72]. It is a self-adhesive material without the need for any pre-treatment procedures. This hybrid cement is self-setting through the reactions of the glass ionomer and acid–base reactions [8]. It has comparable or higher retentive strengths to those of other types of permanent cements [9,20]. CaAl/GIC has sufficient working and setting times, thereby allowing a relatively easy mix process and the removal of excess cement [9,73]. One of the major advantages of this hybrid cement is its bioactivity to promote remineralization [7]. Moreover, the cement provides a negatively charged surface and releases calcium and phosphate ions, thus fulfilling the prerequisites of being bioactive [7]. CaAl/GIC possesses stronger antibacterial properties when it is compared with self-adhesive resin cement and conventional GIC [74]. The sealing ability and microleakage pattern are also comparable to that of self-adhesive resin cement [26]. These characteristics are favorable to inhibiting recurrent caries in patients. In addition, CaAl/GI presents satisfactory postoperative sensitivity, retention, marginal integrity and color stability [72,73,75,76]. However, long-term clinical evaluations are generally lacking, and more laboratory studies comparing its properties such as fluoride release, color stability and mechanical strengths are needed. The indication of CaAl/GIC includes the definitive cementation of metal or porcelain-fused-to-metal crowns and bridges, gold inlays and onlays, cast or prefabricated metal posts, and high-strength ceramic crowns and bridges such as zirconia, alumina and lithium disilicate.

### 4.5. Resin Cements

Both the conventional resin cement with a separate adhesive system (or adhesive resin cement) and the self-adhesive resin cement can be used as luting cements. They have the advantages of high compressive and tensile strengths, a high bond strength, a low water solubility and good aesthetic properties, which make them the leading luting cements today [2]. Proper moisture control is also necessary to achieve a reasonable bond strength for resin cements [77]. In the cases where isolation cannot be achieved, the use of alternative cements may be a better option.

#### 4.5.1. Conventional Resin Cement

Conventional resin cement has a similar chemical composition to restorative resin composites. They consist of resin monomers, such as methyl methacrylate, bisphenol A-glycidyl methacrylate dimethacrylate (Bis-GMA) and urethane dimethacrylate, which undergo polymerization during the setting reaction [5]. The filler concentration in the resin cement is lowered to allow for a thin film thickness and a sufficient working time [6]. The bonding mechanism of conventional resin cement to enamel is made through micromechanical interlocking after acid etching. The removal of the smear layer, surface demineralization and the application of a primer is needed to achieve the resin bonding in dentine [5].

Some conventional resin cements require the prior application of adhesive systems. The adhesive systems of conventional resin cements can be divided into total etch (etch-and-rinse) and self-etch [78] based on the etching mechanism. The biggest disadvantage of the total-etch cement is the multistep application that increases the risk of contamination that compromises the bond strength [79]. There is also a higher risk of postoperative sensitivity when it is compared to the self-etch systems [80]. Moreover, the total-etch strategy only benefits the enamel bonding, but not the dentinal bonding [81]. It has been shown that resin monomers were unable to penetrate into deeper areas of the hybrid layer, resulting in the incomplete infiltration in the dentine [82]. On the other hand, using self-etching adhesives alone reduces the enamel bonding efficacy because the effect of demineralization is less than that which occurs with phosphoric acid [83]. Therefore, selective enamel etching with self-etching adhesives is the suggested strategy to optimize bonding [81].

From the ISO specification 4049 (2019), resin cement can be classified according to the mode of the cure: class 1 (self-cured), class 2 (light-cured), and class 3 (dual-cured). Self-cured cement is used for luting thick, opaque restorations and materials that are not translucent, such as metals and highly opaque ceramics [84]. This is to ensure a high degree of conversion and optimal properties in the areas where light cannot reach [85]. However, this type of cement has the limitations of a reduced working time and the tendency to discolorize due to the high concentration of tertiary amines, which are the polymerization activators [86]. Therefore, in cases where there is a high aesthetic demand such as laminate veneers, a light-cured cement is the best material of choice [87,88].

On the other hand, dual-cured resin cement combines both of the curing modes and has the gain of an increased versatility in its clinical usage. The system contains a catalyst paste with a chemical initiator (benzoyl peroxide) and a base paste with the light-cured resin cement and a tertiary amine [89]. When it is mixed and light-cured, a polymerization reaction occurs by photo and chemical activation. Although the dual-cured cement appears to be the safest choice to ensure sufficient polymerization, the degree of the cure without light activation can be as low as 10.82% [90]. An insufficient light exposure under 4 mm thick ceramic restorations can result in a degree of cure that is even lower than that of the self-cure alone [91]. There have also been studies suggesting extending the light curing to 120s to benefit the degree of conversion and achieve better mechanical strengths [92,93]. Therefore, clinicians should choose the appropriate class of resin cement according to the material and thickness of the restoration to achieve the best outcome. Generally, for ceramic restorations that are thinner than 2.0 mm, light-cured and dual-cured cement produces a better result than self-cured cement does [94].

In general, conventional resin cements are widely indicated for cementing aesthetic all-ceramic and indirect-resin restorations and veneers. The cementation of metal and metal-ceramic restorations with compromised retention and resistance forms, such as resin-bonded bridges and short crowns, and post-cementation in root-treated teeth are also possible indications [2]. Regarding the disadvantages of conventional resin cement, the cost is high, there is a sensitivity to the technique, it is time-consuming, and it is difficult to remove the excess cement. Therefore, it is contraindicated in cementing prefabricated crowns, especially in pediatric patients [51].

#### 4.5.2. Self-Adhesive Resin Cements

Self-adhesive resin cement is a type of resin cement that can adhere to the tooth structures without a separate adhesive and etchant [95]. The first commercial product was developed in the early 2000s to simplify the clinical steps of the application. The major constituents of the self-adhesive resin cement include functional acidic monomers (e.g., 10-methacryloyloxydecyl dihydrogen phosphate), conventional dimethacrylate monomers (e.g., Bis-GMA), fillers and activator-initiator systems [87]. Similar to self-etching adhesive systems, the functional acidic monomers create a low pH and high hydrophilicity during the initial setting reaction. This gives the material the ability to wet and etch the tooth surface to facilitate a homogenous infiltration of the adhesive resin [96]. As the reaction proceeds, the acidic functional groups react with the calcium in the dental tissues and a range of metal oxides from ion-leachable inorganic fillers, resulting in a gradually increased pH and hydrophobicity [97].

Self-adhesive resin cement has the clinical advantage of triggering less postoperative sensitivity when it is compared to RMGIC and GIC [98,99]. However, the adhesive performance and bond strengths to both the enamel and dentine of self-adhesive resin cements are lower than those of conventional multi-step resin cements [100,101]. Although a 2-year study showed that self-adhesive resin cement had similar clinical outcomes to those of the multistep conventional resin cements [102], another 5-year prospective clinical study reported that total-etch resin cement showed a better performance in terms of marginal discoloration and adaptation after the recall [103]. When it is compared to conventional resin cement, the self-adhesive resin cement does not have a separate phosphoric acid etching property in enamel for the wetting effect and creation of microporosities. They only rely on the acidic functional monomers for bonding, which are generally weaker than the conventional etchant is [87]. Moreover, following a tooth preparation by rotary instruments, the enamel and dentine are covered with an acid-soluble smear layer [104]. Because there is no etch-and-rinse procedure before the application, the smear layer is not removed. Although self-adhesive resin cements can interact with the smear layer to reinforce the smear plugs, these structures are micromechanically weaker than the resin tags are and thus, provide a lower bond strength to the dentine [105].

Self-adhesive resin cements provide an improved ease of application than the conventional resin cements can, and they can be used in cementing a variety of indirect restorations. However, because the bond strength is lower than that of the conventional resin cement, it is not recommended to use self-adhesive resin cement to cement restorations with reduced retention and resistance forms, such as resin-bonded bridges and crowns with insufficient heights. Moreover, due to weaker enamel bonding and a greater incidence of discoloration, it is unsuitable to cement veneers as well [87].

## 5. New Development of Luting Cements

### 5.1. Modifications on Currently Available Luting Cements

#### 5.1.1. Modifications on Glass Ionomer Cement

Nanotechnology has been incorporated into the development of GIC to improve its mechanical and physical properties [106]. Nano-modifications of GIC can be achieved by either reducing the size of the glass particles or the addition of nano-sized fillers or bioceramics to gain the potential benefits of an increased mechanical strength and bioactivity [107,108]. Because it was suggested that resizing the glass particles in GIC to nanoscales does not significantly improve the physical properties of the cement [109], the focus of recent developments has been shifted to the incorporation of nano-sized fillers or the addition of nano-hydroxyapatite crystals as fillers. Hydroxyapatite is a naturally occurring form of the mineral calcium apatite (calcium, phosphorus and oxygen) and is one of the major compositions of bone, enamel and dentine [110]. The research has shown that GIC that is modified by nano-hydroxyapatite has an increased mechanical strength [111] and bond strength to dentine [112]. Moreover, the addition of hydroxyapatite to GIC powder can increase the crystallinity of the set GIC, thereby improving the chemical stability and water insolubility of it [112,113]. Studies have also found that nano-hydroxyapatite-modified GIC not only has a higher compatibility than conventional GIC does, but also a greater release of fluoride ions [114,115].

Another modification is the addition of calcium sodium phosphosilicate bioactive glass to GIC. In 1969, Hench introduced the original phosphosilicate bioactive glass as 45S5 Bioglass with a specific chemical composition and weight percentages of silica dioxide, calcium oxide, sodium oxide and phosphorus pentoxide [56,116]. Although the addition of the phosphosilicate bioactive glass to GIC increased the remineralization, the mechanical strength of the material was found to be compromised [117,118,119]. In a recent development, bioactive glass nanoparticles have been used to modify GIC instead of the conventional micro-sized ones. Bioactive glass nanoparticles carry the advantages of phosphosilicate bioactive glass, but with increased compressive, tensile and flexural strengths [120]. However, the long-term effects that they have on the human body are unknown, and further studies are still needed before their clinical application in dentistry.

#### 5.1.2. Modifications on Resin Cement

The recent development of resin cement has been made to enhance its anticariogenic capacity [121]. Despite it being the most widely used luting cements in modern dentistry, resin cement has no antibacterial effect. It cannot prevent recurrent caries, which is one of the main reasons for the failure of the restorations [122]. The modifications of resin cement include the addition of antimicrobial compounds, such as silver nanoparticles, quaternary ammonium polyethyleneimine nanoparticles, cetylpyridinium chloride modified montmorillonite and chlorhexidine diacetate and ursolic acid [123,124,125,126,127]. Efforts have been made to determine the ideal concentrations of these compounds without adversely affecting the mechanical properties of the resin, while also retaining their antibacterial abilities [124].

Some novel developments of resin cements have been made to introduce “self-healing” capacities for the dental resin to resist cracks and fractures. This is achieved by embedding microcapsules with an external shell and healing liquid into the composite material [128,129]. When cracking occurs in the polymer, the microcapsules rupture and release the healing liquid into the crack planes. This exposes them to the catalysts in the polymer matrix, and consequently, the patient undergoes polymerization to fill the crack [129]. A recent development on resin composites using triethylene glycol dimethacrylate-N and N-dihydroxyethyl-*p*-toluidine healing liquid in poly(urea-formaldehyde) shells successfully exhibited a substantial self-healing capability in terms of the virgin fracture toughness [130].

Another direction to modify the resin cement was to enhance the polymerization of the resin monomers because the mechanical properties and bond strengths of the resin cement are affected by the polymerization of the resin monomers. Because the acidic functional monomers inhibit free radical formation by the traditional benzoyl periodize/tertiary amine initiators, researchers have developed the process of chemically curing the co-initiators to achieve a more efficient curing [131]. Another approach to enhance the polymerization of the resin cement is known as “touch curing” or “contact curing” [132]. In this system, a proprietary non-tertiary amine accelerator is present in the primer, which accelerates the curing when it is in contact with the cement [133].

#### 5.1.3. Restorative Composite Resin as an Alternative Luting Material

Recently, there have been studies looking into the possibility of using restorative composite resin as an alternative luting material due to it having a lower cost, better strength properties, lower marginal deterioration, and greater range of shade selections [134]. As a restorative material, composite resin differs from resin cements by having a higher percentage of filler particles, resulting in it having a higher viscosity, greater film thickness and lower flowability [135]. To solve these problems, preheating and ultrasonic vibration techniques were tested to reduce the film thickness and viscosity [136]. Both of the techniques were shown to be effective in reducing the film thickness of the material. However, not all of the tested composite resins after preheating and ultrasonic vibration were able to reach a film thickness of less than 50 μm [134], which is specified in ISO standard 4049:2019 [137]. When a thin film thickness and a high viscosity cannot be achieved, the fracture resistance of the restoration and marginal adaptation will be drastically jeopardized [138,139,140]. Hence, other modification techniques should be studied in the future before the composite resin can be modified to be a luting agent.

### 5.2. Development of Novel Luting Materials—Castor Oil Polyurethane Cement

Castor oil polyurethane is a biomaterial with great biocompatibility, and it is easy to handle. It is extracted from a plant called *Ricinus communis*, which is abundantly found in Brazil [141]. Due to its large commercial availability, it is expected to be a prospective versatile dental material with a reduced cost, and it is highly sustainable. As castor oil polyurethane has good biocompatibility properties, osteo-inductive properties and antimicrobial abilities, it has great potential to be developed as a versatile dental material. Some studies have tested its use as a luting cement [141,142,143]. The luting material was presented as a two-part system with calcium carbonate fillers [142]. Although the flexural strength of castor oil polyurethane was only 25% of that of adhesive resin cement, it is comparable to the conventional cements such as zinc phosphate and GIC [143]. Modifications of castor oil polyurethane with the addition of different fillers to enhance its mechanical strength could be the direction of its future development.

## 6. Summary

Luting material aids in the retention and stability of indirect restorations. The common luting materials include zinc oxide eugenol and non-eugenol, zinc phosphate, zinc polycarboxylate, GIC, RMGIC, resin cement and hybrid CaAl/GI. Each of them possesses unique properties and clinical implications. Because none of the currently available luting materials can fulfil all of the clinical applications, researchers are modifying GIC and resin cements with various technologies and developing novel luting materials, such as castor oil polyurethane cements, to meet various clinical requirements.

## Figures and Tables

**Table 1:** Properties and the types of temporary luting cements.

Properties	Ideal Materials [12]	Zinc Oxide Eugenol	Zinc Oxide Non-Eugenol	Zinc Polycarboxylate
Bond strength	Low (for easy of removal)	Low	Low	Low
Handling properties [13]	Good	Good	Good	Fair (cement is hard to mix)
Ease of cleaning up [13,14]	High	High	High	Low (cement is hard to remove)
Effect on permanent cementation [15,16,17]	No adverse effect	Interfere resin cement	No adverse effect	No adverse effect
Pulpal effect [13,18,19]	Minimal pulpal irritation Sedative	Anti-inflammatory anaesthetic	Minimal pulpal irritation	Minimal pulpal irritation

**Table 2:** Properties and permanent luting cements.

Properties	Ideal Material [4]	Zinc Phosphate	Zinc Poly-Carboxylate	GIC	RMGIC	Hybrid CaAl/GIC	Conventional Resin Cement	Self-Adhesive Resin Cement
Compressive strength (MPa) [20,21]	High	48 (Flecks)	63 (Durelon)	105 (Ketac Cem)	96.3 (RelyX Luting)	160 (Ceramir C&B)	209 (Scotchbond resin cement)	157 (RelyX Unicem)
Elastic modulus (GPa) [21,22]	13.7 (dentine)	19.8 (Flecks)	16.1 (Durelon)	19.5 (Ketac Cem)	6.8 (Vitremer)	No data	11.8 (Scotchbond resin cement)	16.5 (RelyX Unicem)
Shear bond strength ^1^ (MPa) [9,23,24]	High	0.65 (N.A.)	1.40 (N.A.)	2.36 (GC Fuji 9)	2.53 (GC Fuji Plus)	5.79 ^2^ (Ceramir C&B)	6.99 (Panavia F 2.0)	5.07 (Clearfil SA)
Fluoride release	Yes	No	No	Yes	Yes	Yes	No	Yes
Microleakage [12,25,26,27]	Minimal	High	High to very high	Low to very high	Very low	Low to high	Very low	Very low
Film thickness (µm) [12,20,28,29,30,31]	Thin	<25 (N.A.)	<25 (N.A.)	24.2 (GC luting)	25.2 (GC Fuji Plus)	16.4 (Ceramir C&B)	24.3 (Panavia 21)	16.0 (RelyX Unicem)
Working time (min) [12,31,32,33,34]	Long	~2:30 (DeTrey Zinc)	2:00–2:30 (Poly-F Plus)	3:10 (Ketac Cem)	2:30 (GC Fuji Plus)	2:00 (Ceramir C&B)	4:00 (Panavia 21)	2:30 (RelyX Unicem)
Setting time (min) [20,31,32,33,34]	Short	5:00–6:00 (DeTrey Zinc)	5:00–7:00 (Poly-F Plus)	7:00 (Ketac Cem)	4:30 (GC Fuji Plus)	~4:48 (Ceramir C&B)	7:00 (Panavia 21)	6:00 (RelyX Unicem)
Removal of excess [9,12]	Easy	Easy	Medium	Medium	Medium	Easy	Difficult	Medium
Water solubility [12]	Minimal	High	High	Low	Very low	Low	Very low	Very low
Aesthetics	High	Low	Low	Low	Moderate	Low	Highest	High
Color stability	High	Low	Low	Low	Moderate	Low	Highest	High
Pulpal irritation [12]	Low	Moderate	Low	High	High	No data	High	High

^1^: Values based on studies of cementation of zirconia onto dentine. ^2^: Not tested under universal setting. GIC—Glass ionomer cement; RMGIC—Resin-modified glass ionomer cement; Hybrid CaAl/GIC; Hybrid calcium aluminate/glass ionomer cement.

**Table 3:** Suggested clinical uses of luting cements.

Luting Material	ZOE/ZONE	Zinc Phosphate	Zinc Poly-Carboxylate	GIC	RMGIC	Hybrid CaAl/GIC	Conventional Resin Cement	Self-Adhesive Resin Cement
**Provisional restoration**								
Metal	✓		✓	✓	✓			
Bis-acryl	✓		✓					
PEMA	✓		✓					
PMMA	✓		✓					
**Single-unit definitive restoration**								
All-metal crown		✓	✓	✓	✓	✓	✓	✓
Metal-ceramic crown		✓		✓	✓	✓	✓	✓
Zirconia/alumina crown					✓	✓	✓	✓
Lithium disilicate crown						✓	✓	
All metal inlay/onlay		✓		✓		✓	✓	✓
Ceramic inlay/onlay					✓	✓	✓	✓
Ceramic veneer							✓	
Composite veneer							✓	
**Endodontic post**								
Cast metal post		✓		✓	✓	✓		✓
Prefabricated metal post		✓		✓	✓	✓		✓
Fiber post					✓			✓
**Multiple-unit definitive restoration/bridge**								
All-metal (conventional)							✓	✓
Metal ceramic (conventional)				✓	✓		✓	✓
Metal ceramic					✓		✓	✓
Lithium disilicate							✓	
Zirconia							✓	✓
Metal ceramic resin-bonded							✓	
**Orthodontic appliances**								
Bands and brackets				✓	✓			✓

ZOE/ZONE—zinc oxide eugenol/zinc oxide non-eugenol; GIC—Glass ionomer cement; RMGIC—Resin-modified glass ionmer; Hybrid CaAl/GIC—Hybrid calcium aluminate/glass ionomer cement.

## Data Availability

Not applicable.
